# Ca^2+^-stabilized adhesin helps an Antarctic bacterium reach out and bind ice

**DOI:** 10.1042/BSR20140083

**Published:** 2014-07-04

**Authors:** Tyler D. R. Vance, Luuk L. C. Olijve, Robert L. Campbell, Ilja K. Voets, Peter L. Davies, Shuaiqi Guo

**Affiliations:** *Protein Function Discovery Group and the Department of Biomedical and Molecular Sciences, Queen's University, Kingston, Ontario, Canada; †Laboratory of Macromolecular and Organic Chemistry and Institute for Complex Molecular Systems, Department of Chemical Engineering and Chemistry, Eindhoven University of Technology, Eindhoven, The Netherlands

**Keywords:** bacterial Ig-like fold, Ca^2+^-binding, crystal structure, extender domain, ice-binding adhesin, solution structure, aa, amino acid, AFP, antifreeze protein, AUC, analytical ultracentrifugation, BIg, bacterial immunoglobulin, *Mp*AFP, *Marinomonas primoryensis* antifreeze protein, ORF, open reading frame, RDF, radial distribution function, RII, Region II, RII tetra-tandemer, four tandem RII, RIV, repetitive Region IV, RTX, repeats-in-toxin, SAXS, small-angle X-ray scattering, TISS, type I secretion system, WLC, worm-like chain, XRD, X-ray diffraction

## Abstract

The large size of a 1.5-MDa ice-binding adhesin [*Mp*AFP (*Marinomonas primoryensis* antifreeze protein)] from an Antarctic Gram-negative bacterium, *M. primoryensis*, is mainly due to its highly repetitive RII (Region II). *Mp*AFP_RII contains roughly 120 tandem copies of an identical 104-residue repeat. We have previously determined that a single RII repeat folds as a Ca^2+^-dependent immunoglobulin-like domain. Here, we solved the crystal structure of RII tetra-tandemer (four tandem RII repeats) to a resolution of 1.8 Å. The RII tetra-tandemer reveals an extended (~190-Å × ~25-Å), rod-like structure with four RII-repeats aligned in series with each other. The inter-repeat regions of the RII tetra-tandemer are strengthened by Ca^2+^ bound to acidic residues. SAXS (small-angle X-ray scattering) profiles indicate the RII tetra-tandemer is significantly rigidified upon Ca^2+^ binding, and that the protein's solution structure is in excellent agreement with its crystal structure. We hypothesize that >600 Ca^2+^ help rigidify the chain of ~120 104-residue repeats to form a ~0.6 μm rod-like structure in order to project the ice-binding domain of *Mp*AFP away from the bacterial cell surface. The proposed extender role of RII can help the strictly aerobic, motile bacterium bind ice in the upper reaches of the Antarctic lake where oxygen and nutrients are most abundant. Ca^2+^-induced rigidity of tandem Ig-like repeats in large adhesins might be a general mechanism used by bacteria to bind to their substrates and help colonize specific niches.

## Abstract 2

An Antarctic bacterium, *Marinomonas primoryensis*, uses a 1.5-MDa adhesin to bind ice. Crystal and solution structures of four of its 120 tandem Ig-like repeats show how Ca^2+^ rigidifies and projects the adhesin from the bacteria to the ice.

## INTRODUCTION

RTX (repeats-in-toxin) proteins are a family of Ca^2+^-binding proteins produced by Gram-negative bacteria [1]. They are exported via the TISS (type I secretion system) and are involved in a wide range of biological functions. First discovered as pore-forming toxins, RTX proteins have subsequently been characterized as bacterial lipases, proteases, and S-layer forming proteins [1,2]. Recently, RTX proteins of a novel subtype have been classified as high molecular mass repetitive adhesion proteins, which are often encoded by the largest genes (>6000 nucleotides) of the bacterial genomes. These extremely large adhesins typically include many (>25) tandem repeats of an 80–120-residue domain near the N-terminus that account for the majority of the protein's mass. Several 9-residue Ca^2+^-binding RTX repeats (typically GGxGxDxUx, where x can be any residue and U is a hydrophobic residue) occur close to the C-terminus. The RTX adhesins help form multicellular communities, and their interactions with various surfaces allow bacteria to colonize and infect-specific niches. Some of the well-characterized RTX adhesins include biofilm-associated proteins such as LapA [8682 aa (amino acid)] and LapF (6310 aa) from *Pseudomonas putida* [2–4]; and epithelial-cell adhesins that contribute to pathogenesis such as SiiE (5559 aa) from *Salmonella enterica* and FrhA (2821 aa) from *Vibrio cholera* [5,6].

A 1.5-MDa RTX adhesin [*Mp*AFP (*Marinomonas primoryensis* antifreeze protein)] with ice-binding activity was found on the surface of the Gram-negative bacterium, *Marinomonas primoryensis*, from Antarctica [7–9]. *Mp*AFP can be divided into five distinct Regions (RI–RV) that include the highly repetitive RII (Region II) and the moderately repetitive RIV (Region IV). The 322-aa RIV is solely responsible for the ice-binding activity of *Mp*AFP [8,10], and its crystal structure reveals thirteen RTX repeats that each bind a Ca^2+^ to fold the domain into a β-solenoid [11]. RII consists of approximately 120 tandem copies of a perfect 104-aa repeat that account for over 90% of the mass of the 1.5-MDa protein. We recently solved the X-ray crystal structure of a single 104-aa RII repeat (referred to here as a tandemer) to 1.35-Å resolution [12]. The RII-tandemer is a BIg (bacterial immunoglobulin)-like beta-sandwich domain that requires at least three Ca^2+^ ions for folding. Ca^2+^ ions were also coordinated at the interfaces between the RII-tandemer and its symmetry-related neighbours within the crystal that helped individual BIg domains interact in a head-to-tail fashion. This observation suggested that Ca^2+^ might play a role in strengthening and extending the massive tandem array of the RII domains to form a rigid rod-like structure. We hypothesized that *Mp*AFP_RII serves as a Ca^2+^-dependent extender domain to project the ice-binding RIV away from other cell surface molecules in order to bind *M. primoryensis* to ice. The selective advantage of having this adhesin would be to help the strictly aerobic *M. primoryensis* remain in the upper reaches of the ice-covered Antarctic lake where oxygen and nutrients are most abundant.

To gain insight into the overall architecture of the ~120 tandem RII domains, we set out to produce, crystallize and determine the 3-D structure of a RII segment spanning four tandem repeats. Here we report the 1.8 Å-resolution crystal structure of the RII tetra-tandemer. It shows how the four RII repeats fold into a rigid and elongated structure in the presence of Ca^2+^. We used SAXS (small-angle X-ray scattering) to demonstrate the RII tetra-tandemer (four tandem RII) is significantly rigidified in the presence of Ca^2+^, and that its solution structure is in excellent agreement with the crystal structure. Using a combination of CD, size-exclusion chromatography and AUC (analytical ultracentrifugation) we show Ca^2+^ is indispensable for folding and rigidifying the structure of the tandem RII domains. We suggest the Ca^2+^-induced rigidity in the large repetitive extender domains of RTX adhesins is a general mechanism used by Gram-negative bacteria, including pathogens, to bind to their specific substrates.

## MATERIALS AND METHODS

### Construct design and cloning of the RII tetra-tandemer gene

**Figure 1:**
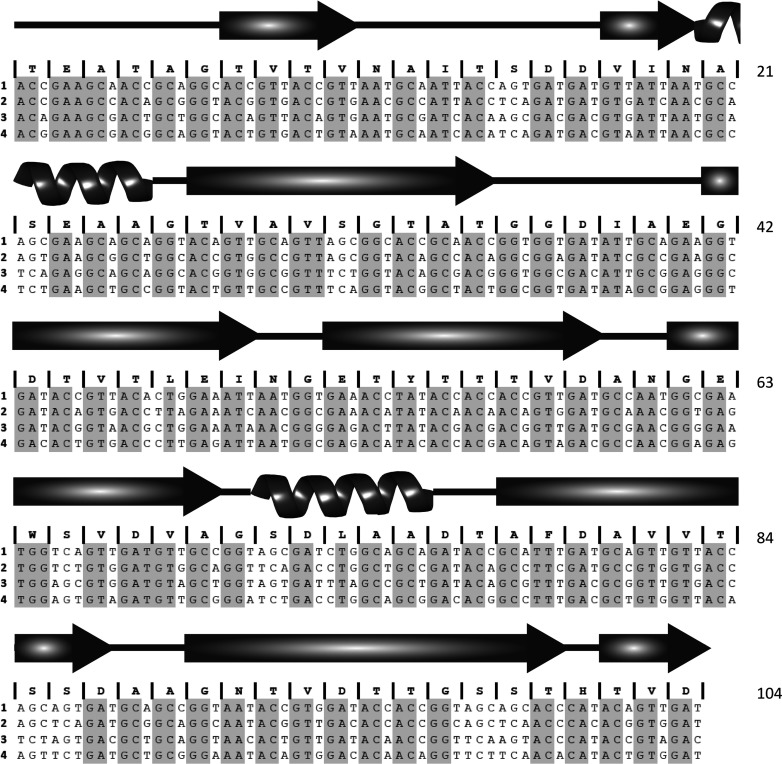
Altered DNA sequences coding for the four Ig-like domains in the RII tetra-tandemer synthetic gene The DNA sequences for each of the four repeats (1–4) are aligned, with the conserved aa shown above each codon. Identical nucleotides among all four repeats are highlighted in grey. The corresponding secondary structure is shown above. Residue numbers are shown on the right.

The DNA construct of the RII tetra-tandemer was synthesized by GeneArt (Life Technologies). The four tandem 312-bp repeats were codon-optimized for *Escherichia coli* expression using codon degeneracy while making each repeat as distinct as possible at the DNA sequence level to lessen the chances of recombination (Figure 1). No changes were made to the original aa sequence. Additionally, the G–C content of the DNA sequence was optimized to minimize the formation of RNA secondary structure that could hamper translation. The construct was inserted between *Nde*I and *Xho*I sites in the pET-28a expression vector. Positive clones were identified by restriction digestion and DNA sequencing (Robarts Research Institute, London, Ontario, Canada).

### Expression and purification of the RII tetra-tandemer

Positive clones were electroporated into the *E. coli* BL21DE3 (star) expression cell line. A 1-L culture was grown in the presence of 100 μg/ml kanamycin at 37°C with shaking until the *A*_600_=0.6. The culture was then switched to 23°C until the *A*_600_=0.9, whereupon protein production was induced by the addition of 1 mM IPTG (isopropyl β-D-thiogalactoside) and growth was continued overnight at 23°C with shaking. The cell pellet was recovered by centrifugation and lysed by sonication in buffer containing 50 mM Tris–HCl (pH 9), 500 mM NaCl, and 2 mM CaCl_2_. Cellular debris and insoluble matter were removed by centrifugation for 0.5 h at 16000 rpm in a JA25.5 rotor. The N-terminally 6× His-tagged protein was selected from other proteins by Ni-NTA affinity chromatography. The RII tetra-tandemer was then buffer-exchanged into a solution of 50 mM Tris–HCl (pH 9), 200 mM NaCl and 10 mM CaCl_2_ using a centrifugal filter (Millipore). Concentrated protein was loaded onto a HiLoad 16/60 Superdex-200 size-exclusion column (GE Healthcare) for further purification. Fractions containing the tetra-tandemer were pooled and stored at 4°C for future use. Protein concentration was measured with a Nanodrop spectrophotometer (Thermal Fisher Scientific) and the purity was assessed by SDS/10%PAGE.

### Size-exclusion asymmetry assay

Samples containing RII tetra-tandemer (0.8 mg) were mixed with EDTA/CaCl_2_ to produce five solutions of the following concentrations: 0.5 mM EDTA, 0 mM CaCl_2_, 4 mM CaCl_2_, 10 mM CaCl_2_ and 20 mM CaCl_2_. Each solution was loaded on to a 10/300 GL Superdex-200 size-exclusion column (GE Healthcare) and eluted using a running buffer of the same CaCl_2_/EDTA concentration in 50 mM Tris–HCl (pH 9) and 200 mM NaCl. The elution volume of the tetra-tandemer in each solution was compared with those of the protein standards, in order to deduce the apparent molecular mass. The void volume (*V*_0_) was determined from the elution of blue dextran; the column volume (*V_t_*) was marked by the elution of NaCl.

### Analytical ultracentrifugation

Sedimentation velocity measurements in a Beckman Optima XL-I Analytical ultracentrifuge (Beckman Coulter) were done using double sector charcoal-Epon cells equipped with quartz windows and were performed at 20.0°C on 0.68 mg/ml samples in 50 mM Tris–HCl (pH 9.0), 20 mM NaCl with either 2 mM CaCl_2_ or 0.5 mM EDTA. Concentration distributions were determined by sedimentation velocity at 40000 rpm using absorbance optics. Sedimentation coefficient distributions were determined using the program SEDFIT, which fits the sedimentation velocity data directly to the Lamm equation and uses mathematical methods to obtain a numerical solution to this equation [13]. SEDNTERP was used to calculate the partial specific volume (0.71 ml/g) and the buffer density 1.01 g/ml and viscosity (0.01 P).

### CD and calcium titration

RII tetra-tandemer was dialysed against buffer containing 5 mM Tris–HCl (pH 9) and 0.1 mM EDTA. A subsequent dilution with additional buffer was performed to lower the protein concentration to 8 μM. Individual aliquots of RII tetra-tandemer were then mixed with CaCl_2_ to produce 4: 1, 20: 1, 40: 1 and 80: 1 molar ratios of CaCl_2_/RII tetra-tandemer. Samples were scanned at 23°C using a Chirascan CD Spectrometer (Applied Photophysics), with seven scans collected, averaged and buffer reference-subtracted for each. Three-point smoothing using PROVIEWER software was then applied. Deconvolution of the spectra was performed with OLIS SpectralWorks (On-Line Instruments).

### Crystallization, data collection and structure determination

Initial crystals were obtained using microbatch methods. The RII tetra-tandemer was buffer-exchanged into 20 mM Tris–HCl (pH 9) and 10 mM CaCl_2_ and concentrated to 15 mg/ml. Equal volumes (1 μl) of the protein solution and a series of high Ca/Mg precipitant solutions were mixed and allowed to equilibrate under a layer of 100% Paraffin Oil. Wells containing 0.2 M calcium chloride, 0.1 M MES (pH 6) and 20% (w/v) PEG 6000 yielded multicrystalline masses that formed at room temperature in approximately 2 days. Crystals suitable for structure determination were obtained using microbatch methods by mixing equal volumes (2 μl) of 15 mg/ml RII tetra-tandemer with the same precipitant solution as above, followed by the addition of 0.5 μl of 5% (w/v) *n*-Octyl-β-D-glucoside.

Crystallization occurred at room temperature with long plate-like crystal clusters appearing after 2 days. Single long plate-like crystals were released from the clusters using a fine needle (Hampton Research). Prior to data collection, the crystal was flash-frozen in a cryo solution of 20% (v/v) ethylene glycol and 80% (v/v) of the precipitant solution. Data were collected at the X6A beamline of the National Synchrotron Light Source (Brookhaven National Laboratory) and were indexed and integrated with XDS [14], and scaled with CCP4-Aimless [15,16]. The structure was solved by molecular replacement with CCP4-Phaser [16,17], using the RII-tandemer structure as the search model (PDB: 4KDV) [12]. The initial model of the RII tetra-tandemer was built using CCP4-Buccaneer [16,18] and was manually corrected in Coot [19]. The structure of the RII tetra-tandemer was refined with the CCP4-Refmac5 [16,20], and Phenix-refine using the simulated annealing and TLS options [21–23].

### SAXS data acquisition and reduction

SAXS data were collected on a Ganesha lab instrument (SAXSLAB) equipped with a GeniX-Cu ultra-low divergence source producing X-ray photons with a wavelength of 1.54 Å and a flux of 10^8^ ph/s. The scattering intensity was measured as a function of momentum transfer vector *q*=4π (sinθ)/λ, where λ is the radiation wavelength and 2*θ* is the scattering angle. Three sample-to-detector distances of 113, 713 and 1513 mm were used to cover an angular range of 0.006<*q*<2.41 Å^−1^.

Samples were measured in polycarbonate (ENKI, KI-Beam) capillaries with a diameter of *d*=2 mm kept in a temperature-controlled holder at *T*=20°C. The 2D scattering data were recorded on a Pilatus 300 K silicon pixel detector with 487×619 pixels of 172 μm^2^. The beam centre and *q*-range swere calibrated using a silver behenate standard. Two-dimensional SAXS patterns were brought to absolute intensity scale using the calibrated detector response function, known sample-to-detector distances, and measured incident and transmitted beam intensities. These normalized SAXS patterns were subsequently azimuthally averaged to obtain the 1D SAXS profiles. Data were collected at protein concentrations of 5 and 20 mg/ml and subsequently merged. The merging of SAXS profiles is customary to generate a profile of sufficient signal-to-noise in the entire *q*-range. This is required for subsequent data analysis without introducing interference effects due to non-negligible protein–protein interactions [as *S*(*q*) deviates from unity], which becomes more prominent at low *q* values and elevated concentrations. The normalized background scattering profile of the buffer and polycarbonate cell was subtracted from the normalized sample scattering profiles to obtain the protein scattering curve. The absolute scale calibration of the scattering curves was verified using the known scattering cross-section per unit sample volume, dΣ/dΩ, of water, being dΣ/dΩ (0)=0.01632 cm^−1^ for *T*=20°C [24,25].

### Data analysis

All SAXS data processing steps, such as solvent subtraction and data merging, were performed using PRIMUS from the ATSAS software package [26]. The experimental 1D scattering profiles were analysed using a Guinier approximation to extract the radius of gyration (*R_g_*) and the forward scattering intensity (*I*_0_), where *I*_0_=dΣ/dΩ(*q*◇0), which is valid for monodisperse spherical particles at small angles (*q*≤1.3/*R_g_*). The forward scattering intensity *I*_0_ was used to calculate the molar mass of the protein (Supplementary Table S1at http://www.bioscirep.org/bsr/034/bsr034e121add.htm) [25]. Furthermore, the scattering profiles were analysed using a form factor for self-avoiding WLCs (worm-like chains) [27], which is implemented in the software package SASview. Information on the dimensions of the proteins was extracted assuming a uniform scattering length density along the cross-section (see the Supplementary data at http://www.bioscirep.org/bsr/034/bsr034e121add.htm for more information).

### Molecular shape reconstruction

The *ab initio* molecular shape of the protein in solution was reconstructed using simulated annealing methods implemented in DAMMIN [28]. First, an inverse Fourier transformation was applied to the experimental scattering data to obtain the RDF (radial distribution function), describing the probability of finding interatomic vectors of length (*r*) within the scattering particle, using GNOM [29]. The maximum linear dimension (*D*_max_) was set to approximately 3*R_g_ and adjusted to give the best fit to the experimental data. The RDF was considered to be zero at *r*=0 Å and approaches zero at *D*_max_. The GNOM output files were used as input for the simulated annealing calculations using DAMMIN. Ten independent dummy atom models were calculated from a predefined cylindrical shape with radius 25 Å and length 200 Å, without point symmetry (P1). The ten different models were aligned using DAMSEL followed by DAMSUP, and averaged using DAMAVER to compute the probability map [30]. Finally, DAMFILT was used to filter the averaged model to give a structure that has high densities on the probability map representing the molecular shape of the protein in solution.

## RESULTS

### Construction of the RII tetra-tandemer

RII is made up of ~120 Ig-like β-sandwiches that are identical at the DNA level. When PCR primers complementary to the beginning and end of the RII-repeat were used in attempts to amplify a series of multiple repeats the yield of PCR products longer than two repeats in length was too low to extract DNA for cloning (results not shown). Also, with perfect repeat identity comes the potential for recombination once the DNA is in *E. coli* that could lead to deletions within the tandem repeats [31].

To circumvent problems with amplification by PCR the gene was synthesized. To avoid recombination the DNA sequence of four identical repeats was altered through codon degeneracy to produce four domains in tandem that, while maintaining 100% sequence identity at the protein level, possessed a sequence identity at the DNA level of ~70%. The aligned DNA sequences for each of the four altered repeats are shown alongside the secondary structure notations (Figure 1). The cache of potential codons for each residue was limited by the expression preference of *E. coli* for certain codons as well as the need to prevent RNA secondary structure that could impair translation. Therefore the final construct was a compromise between codon optimization, G–C content and sequence non-identity at the DNA level.

### RII tetra-tandemer is monodisperse and has an extended conformation in the presence of Ca^2+^

**Figure 2:**
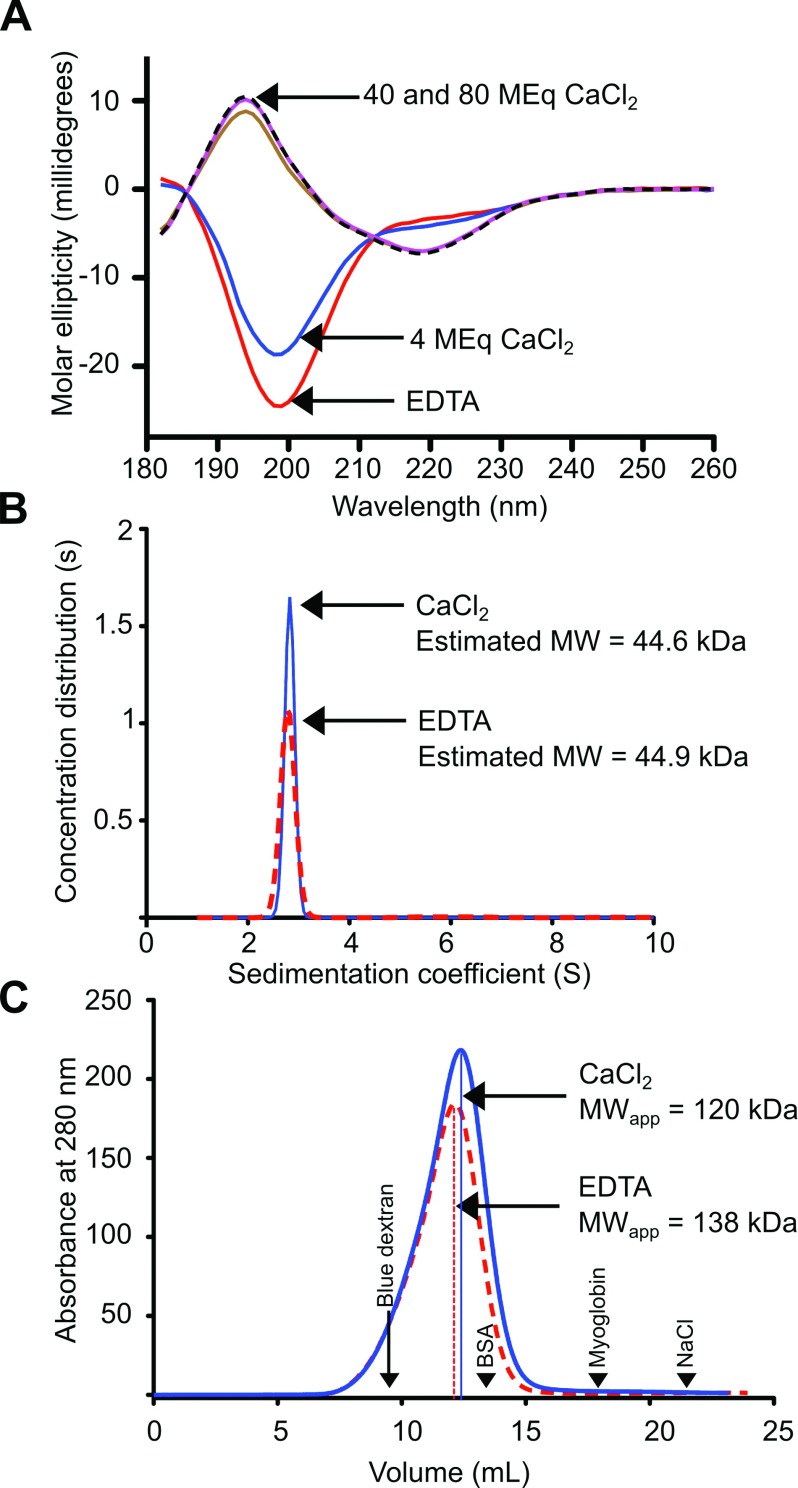
Biophysical analysis of the RII tetra-tandemer in Ca^2+^ and EDTA (**A**) Far-UV CD spectra of the RII tetra-tandemer were plotted as molar ellipticity versus wavelength. The CD spectrum in the presence of 0.1 mM EDTA is indicated by a red line. The CD spectra in the presence of 4, 20, 40 and 80 MEq of CaCl_2_ with respect to the RII tetra-tandemer are indicated by blue, brown, pink continuous lines and a black hatched line, respectively. Arrows point to the red trace at the bottom, the blue trace in the middle, and the pink and black-hatched traces at the top. (**B**) Sedimentation coefficient distributions of the RII tetra-tandemer in the presence of 2 mM CaCl_2_ (blue line), and in 0.5 mM EDTA (red-hatched line). Arrows point to the peaks of the EDTA and CaCl_2_ profiles, respectively. (**C**) Determination of apparent molecular mass by Superdex-200 size-exclusion chromatography. Absorbance at 280 nm was plotted against elution volume for the RII tetra-tandemer. The blue line indicates the chromatogram of the RII tetra-tandemer in the presence of 4 mM CaCl_2_, whereas the red hatched line indicates the chromatogram of the same protein in the presence of 2 mM EDTA. The elution volumes of protein standards (BSA, 67 kDa; myoglobin, 17 kDa) as well as sodium chloride (total volume) and Blue dextran (void volume) are indicated (black arrows).

We have previously shown that the RII-tandemer is fully structured in 10 molar equivalents of Ca^2+^ but resembles a random coil in the absence of this ion [12]. Similar analyses were applied to the RII tetra-tandemer. In the presence of EDTA, the RII tetra-tandemer appeared to be unstructured with its far-UV CD spectrum displaying a single negative peak at 198 nm (Figure 2A). When the CD spectrum was recorded at a 4:1 molar ratio of CaCl_2_/RII tetra-tandemer, an isodichroic point appeared at ~210 nm, indicating a change in the protein's conformation. The RII tetra-tandemer measured at five times this CaCl_2_ concentration (20 molar equivalents) displayed a strong positive peak at 194 nm and a broad negative peak at ~218 nm, which was similar to spectra obtained from proteins rich in β-sheets. The spectra recorded for the RII tetra-tandemer at 40 and 80 molar equivalents of CaCl_2_ were nearly identical, suggesting the protein was fully folded as a β-rich structure at a 40-fold molar ratio of CaCl_2_.

To investigate the oligomeric state of the RII tetra-tandemer in solution, the molecular mass (MW) of the protein was determined by AUC in a sedimentation velocity experiment. The measurement was carried out at 20°C with ~1.2 mg/ml RII tetra-tandemer in the presence of 2 mM CaCl_2_. The data showed a close fit to a single species, with randomly distributed residuals and a low variance (±0.5%, not shown). When the concentration distribution was plotted as a function of sedimentation coefficient, it displayed a single large peak with an estimated molecular mass of 44.6 kDa (Figure 2B). As the calculated molecular mass (MW_act_) of the RII tetra-tandemer is 42.5 kDa (without Ca^2+^), the result indicated the single species observed was the RII tetra-tandemer in its monomeric form. The MW of the RII tetra-tandemer determined by AUC in the presence of 0.5 mM EDTA was 44.9 kDa, which showed a negligible difference compared with the estimated MW in CaCl_2_.

The above sedimentation velocity analyses also provided an estimate of protein shape asymmetry. The frictional ratio (*f*/*f*_o)_ of the monomeric RII tetra-tandemer, where *f* is the translational frictional coefficient of the protein, and *f*_o_ is the theoretical coefficient for a spherical protein of the same mass was calculated to be 1.8 and 2 in the presence of Ca^2+^ and EDTA, respectively, indicating a high level of asymmetry in the protein's conformation [32].

The asymmetry of the RII tetra-tandemer was also assessed by size-exclusion chromatography, which was used to determine the protein's apparent molecular mass (MW_app_). In the presence of CaCl_2_, the RII tetra-tandemer eluted from a calibrated S-200 column with an MW_app_ of ~120 kDa (Figure 2C), which is roughly three times the protein's MW_act_ (42 kDa). Since results from CD and AUC indicated that the RII tetra-tandemer is fully structured in its monomeric form in a Ca^2+^-containing solution, the high MW_app_ of the protein indicates that the protein has a greatly extended shape. The MW_app_ of the RII tetra-tandemer was even larger (138 kDa) in the presence of 0.5 mM EDTA, which is to be expected if the protein was partially unfolded. The MW_app_ of the RII tetra-tandemer decreases slightly with an increase in Ca^2+^ concentration (Table 1), suggesting that the divalent metal cation helps the protein form a more compact and rigid conformation.

**Table 1:** MW_act_, MW_app_ and *V_e_*/*V_t_* values calculated for the protein standards and RII tetra-tandemer Note: *V_e_* for blue dextran indicates the void volume (*V_o_*), whereas *V_e_* for NaCl indicates the total volume (*V_t_*) of the Superdex-200 size-exclusion column. NA, not applicable.

Protein/salt	MW_act_ (kDa)	MW_app_ (kDa)	***V_e_*/*V_t_***
NaCl	NA	NA	1.000
Blue dextran	2000	NA	0.431
Amylase	200	170	0.523
BSA	67	84	0.595
Myoglobin	17	16	0.764
RII tetra-tandemer (EDTA)	42	138	0.545
RII tetra-tandemer (0 mM Ca)	42	138	0.545
RII tetra-tandemer (4 mM Ca)	42	122	0.557
RII tetra-tandemer (10 mM Ca)	42	121	0.558
RII tetra-tandemer (20 mM Ca)	42	118	0.560

### Crystal structure of RII tetra-tandemer reveals a Ca^2+^-dependent extended chain of Ig-like β-sandwich domains

**Figure 3:**
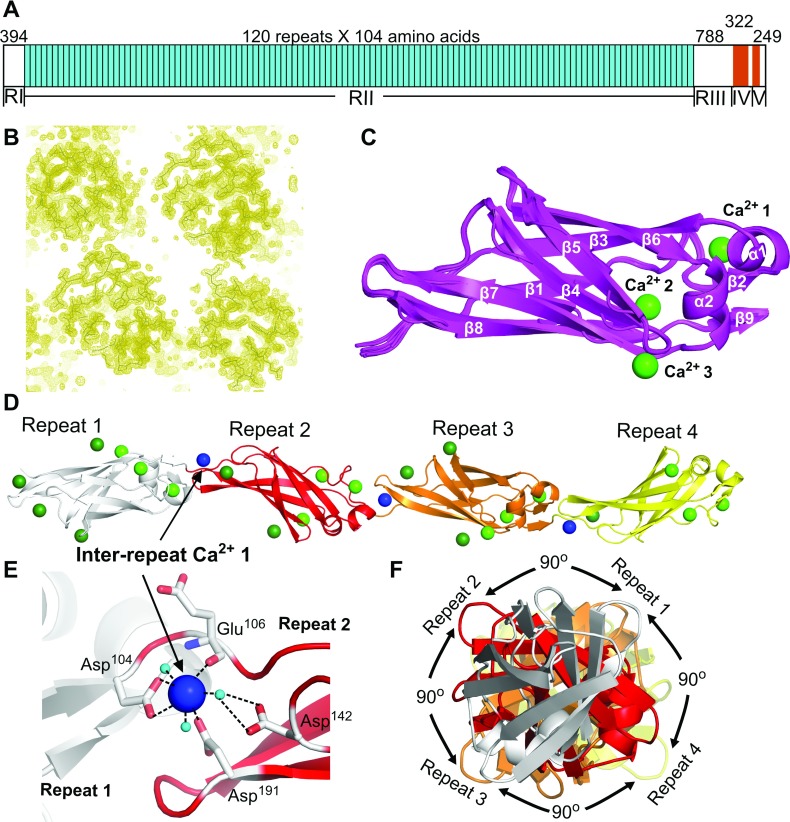
Structure of *Mp*AFP_RII tetra-tandemer (**A**) *Mp*AFP consists of five distinct domains (I–V). The ~120 tandem 104-aa RII repeats are coloured cyan. The RTX repeats in the C-terminal region (RIV and RV) are illustrated as brown blocks. (**B**) 2Fo–Fc electron density of the unit cell contoured at 1σ showing four copies of the RII tetra-tandemer. (**C**) Structural alignment of the 16 RII-repeats within the unit cell. Each individual RII repeat is shown in cartoon representation and is coloured magenta. The three conserved Ca^2+^ ions with full site occupancy are shown as light green spheres. (**D**) Cartoon representation of the RII tetra-tandemer. Ca^2+^ ions are shown as spheres. The intra-RII-repeat Ca^2+^ are coloured light or dark green, while the inter-RII-repeat Ca^2+^ are coloured blue. The four RII-repeats from N- to C-terminus are coloured grey, red, orange and yellow, respectively. (**E**) Enlarged view of the Ca^2+^ that is coordinated in the linker region between RII-repeats 1 and 2. The side chains and main chains of the Ca^2+^-coordinating residues are shown in stick representation. Hatched lines indicate hydrogen bonds. Oxygen atoms are in red, nitrogen atoms are in blue, and water molecules are shown as small aqua spheres. (**F**) Head-on view of a RII tetra-tandemer. The colour scheme is the same as in (**D**).

The crystal structure of the RII tetra-tandemer from *Mp*AFP (Figure 3A) was solved to a resolution of 1.8 Å by the molecular replacement method using the RII-tandemer (PDB: 4 KDV) as the search model. The electron density map was well defined, and over 95% of the residues were automatically built using Buccaneer from CCP4. The RII tetra-tandemer is roughly 190 Å long and 23×28 Å in cross-section. Four copies of the RII tetra-tandemer are packed in the unit cell of the crystal, each oriented antiparallel to its two neighbouring molecules (Table 2; Figure 3B). There are 104 Ca^2+^ ions bound to the four RII tetra-tandemers within the unit cell of the crystal, with a minimum of 24 Ca^2+^ binding to each tetra-tandemer binding. Each individual 104-aa repeat of the RII tetra-tandemer folds as a Ca^2+^-dependent Ig-like β-sandwich that contains seven antiparallel and two short parallel β-strands, and two short α-helices (Figure 3C). Seven β-strands (β1–β6 and β9) and the two α-helices (α1 and 2) help form the compact core region of the Ig-like domain, whereas β7 and β8 comprise a β-hairpin that protrudes from the core, and points toward the N-terminal end of the structure. Structural alignments of the 16 Ig-like domains within the unit cell using PyMOL produced a root-mean-square deviation of 0.27 Å (± 0.09), indicating minimal conformational differences between the RII repeats.

**Table 2:** Diffraction data collection and refinement statistics of the RII tetra-tandemer

Parameter	
Data collection	Dataset
Space group	P1
Cell dimensions	
(a, b, c) (Å)	47.46, 47.47, 191.16
(α, β, γ) (°)	90.04, 90.01, 90.02
Resolution (Å)	47.47–1.80 (2–1.8)
Number of molecules/asymmetric unit	4
*I*/σ*I*	7 (1.49)
*R*_meas_	0.16 (0.92)
CC (1/2)	98.7 (58.4)
Completeness	0.94 (0.93)
Redundancy	2
Refinement	
Resolution (Å)	47.47–1.8 (1.82–1.8)
Number of reflections	148953
*R*_work_/*R*_free_ (%)	22.2/25.7
Number of atoms	
Protein/ligand/water	11301/348/1907
B-factors (Å^2^)	
Protein/ligand/water	23/35.9/27.8
RMS deviations	
Bond lengths (Å)	0.018
Bond angles (°)	1.443

We have previously identified three Ca^2+^ ions that appear to be essential for stabilizing the fold of a single RII repeat (light green spheres, Figures 3C and 3D). These three intra-repeat Ca^2+^ ions all have high occupancies (0.9 or 1) and their coordinations are conserved throughout all individual RII repeats within the unit cell of the RII tetra-tandemer. All other intra-RII-repeat Ca^2+^ are weakly bound to the protein with partial occupancies (~0.5), and seem to play no significant roles in folding the Ig-like domain.

The four tandem Ig-like β-sandwiches of the RII tetra-tandemer are aligned in a highly extended fashion. Each repeat is rotated by approximately 90° relative to its neighbour(s) (Figure 3F), forming an internal 4-fold symmetry within the RII tetra-tandemer. Ca^2+^ ions are also coordinated at the linker regions between the neighbouring repeats. For instance, the inter-repeat Ca^2+^ 1 is hepta-coordinated by three water molecules and four protein ligands from Repeats 1 and 2 (Figure 3E). The Ca^2+^ ion binds to two side-chain oxygen atoms from Repeat 1′s C-terminal Asp^104^, and two oxygen atoms contributed by the main chain of Glu^106^ and the side chain of Asp^191^ from Repeat 2. Moreover, the inter-repeat Ca^2+^ 1 and Asp^142^ from Repeat 2 interact through coordinating a water molecule. Thus the inter-repeat Ca^2+^ mediates the interaction between the tandem RII domains by keeping the C-terminal end of one repeat in close proximity to the β-hairpin (β7 and 8) from the subsequent repeat. As a result of the Ca^2+^-induced rigidity in the linker region, the β-hairpin protruding from Repeat 2 can also interact with Repeat 1 through an extensive network of hydrogen bonding (Supplementary Figure S1 at http://www.bioscirep.org/bsr/034/bsr034e121add.htm). All other inter-repeat Ca^2+^ ions throughout the RII tetra-tandemer are coordinated in a similar way as inter-repeat Ca^2+^ 1.

### SAXS analysis indicates the RII tetra-tandemer is a rigid rod in the presence of Ca^2+^

**Figure 4:**
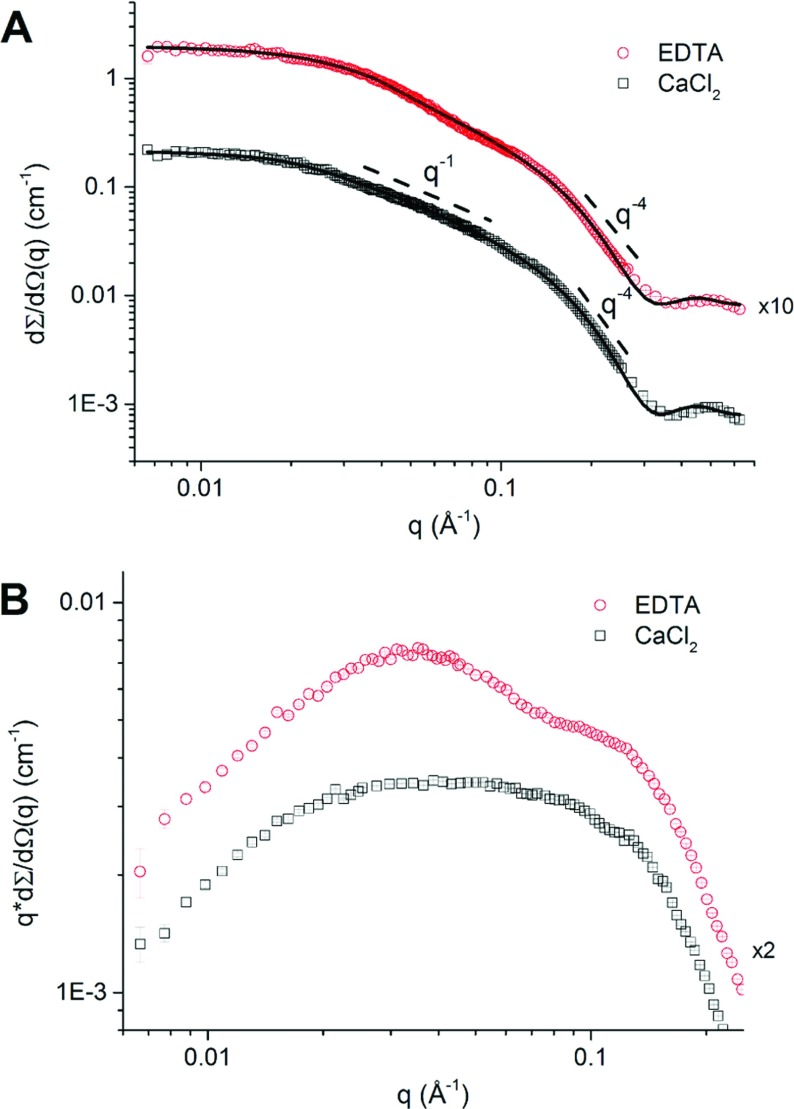
SAXS profiles of *Mp*AFP_RII tetra-tandemer in presence and absence of calcium (**A**) Experimental SAXS data (symbols) and fits with the Schurtenberger–Pedersen form factor for worm-like, self-avoiding chains (solid lines). Dashed lines are drawn to indicate the slope of the scattering curves in the intermediate and high *q-*regimen. The RII tetra-tandemer (squares, circles) was investigated in two buffers composed of (**Ca^2+^**) 20 mM Tris–HCl pH 9, 100 mM NaCl and 20 mM CaCl_2_ and (**EDTA**) 20 mM Tris–HCl pH 9, 100 mM NaCl and 0.5 mM EDTA, respectively. (**B**) Holtzer–Cassasa representation (q*d*Σ/*d*Ω*(*q*) versus *q*) clarifying the change in shape of the scattering curve evident from the difference in length of the Holtzer plateau at intermediate *q*-regimen in 0.5 mM EDTA (circles) or 20 mM CaCl_2_.

SAXS measurements were performed on solutions of the RII tetra-tandemer in buffer with either 20 mM CaCl_2_ or 0.5 mM EDTA. The experimental scattering profiles presented in Figure 4(A) range from the Guinier regimen at low *q*-values up to the first form factor oscillation at high *q*-values. Three power-law regimens are apparent in the SAXS profile recorded in the presence of Ca^2+^. First, a Guinier plateau occurs at low *q* values; at intermediate *q* values the intensity falls off with *q*^−1^, which is typical for rigid 1D objects; and finally at high *q* values the Porod regimen holds where *I* ∝ *q*^−4^. The *I* ∝ *q*^−1^ regimen is much shorter in the presence of EDTA and is preceded by a short power-law regimen with a scaling exponent 1≤α≤2 indicating a considerable reduction in stiffness upon the addition of EDTA. In Figure 4(B), the data are visualized in a Holtzer-Cassasa plot of q*d*Σ/*d*Ω*(*q*) versus *q* to highlight these differences between the samples with EDTA and Ca^2+^ in the intermediate *q*-regimen. The Holtzer–Cassasa representation clearly reveals the Ca^2+^-induced rigidification of the RII tetra-tandemer as evidenced by the differences in the length of the Holtzer plateau in the intermediate *q*-regimen. In line with the CD data (Figure 2A), it is evident from the SAXS profiles that the RII tetra-tandemer undergoes a significant change in fold upon calcium binding. (see also Supplementary Figure S2 at http://www.bioscirep.org/bsr/034/bsr034e121add.htm)

Next, we analysed the experimental data using a form factor originally developed for semi-flexible, self-avoiding polymer chains, which is the WLC model as reported by Schurtenberger and Pedersen [33]. This WLC model describes the conformation of an intrinsically flexible cylinder built up from *N* rigid segments with a related Kuhn length *L_k_*, which is equal to twice the so-called persistence length, *L_p_*. The contour length *L_c_* is then given by the number of locally stiff segments *N* multiplied by their length *L_p_*. The structural parameters obtained from the form factor analysis are given in Table 3. For the RII tetra-tandemer we may compare these to the dimensions computed from the crystal structure obtained by XRD (X-ray diffraction) that show the protein is a rod-like object with a length *L* ~190 Å composed of four rigid subunits of approximately 23 Å×28 Å in cross-section and 45 Å long. We find a good agreement between the XRD and SAXS data for the RII tetra-tandemer in the presence of calcium: application of the WLC model gives *L_c_* ~176 Å, a cross-sectional radius *R_cs_* ~11 Å and persistence length *L_p_* ~95 Å. Here, *L_p_* is larger than the size of one subunit suggesting the formation of a rigid protein complex. Similar to the results obtained from the size-exclusion chromatography experiments (Figure 2C), the RII tetra-tandemer appears larger and less rigid in the presence of EDTA as observed from the increase in contour length *L_c_*~199 Å and decrease in persistence length *L_p_*~41 Å. The persistence length in the presence of EDTA is comparable with the length of one subunit (~45 Å), suggesting that the protein loses its rigidity if no calcium is complexed to the structure.

**Table 3:** Structural parameters obtained from fitting the experimental data of RII tetra-tandemer with a form factor describing a WLC with excluded volume interactions with a circular cross-section of uniform scattering length density given by Schurtenberger and Pedersen [27] *L_c_*=contour length, *L_p_*=persistence length, *R*_cs_=cross-sectional radius of cylinder.

	*L_c_* (Å)	*L_p_* (Å)	*R*_cs_ (Å)
CaCl_2_	175.9±1.8	96.3±2.3	11.2±0.1
EDTA	198.9±0.2	41.2±0.1	11.2±0.1

### Solution structure of the RII tetra-tandemer is in excellent agreement with its crystal structure

**Figure 5:**
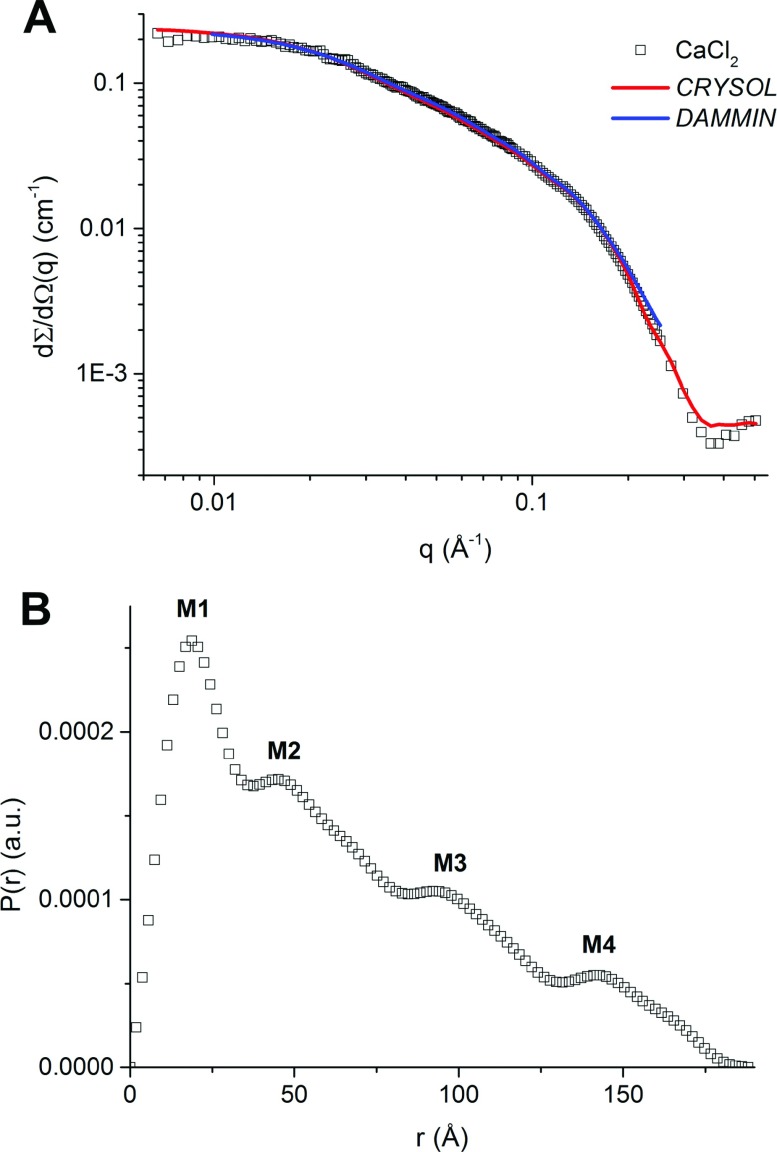
Fit of low-resolution model and crystal structure of RII tetra-tandemer to experimental SAXS data (**A**) Experimental scattering data of RII tetra-tandemer Ca^2+^ (symbols), fit result of *ab initio* modelling (DAMMIN, blue line) and theoretical scattering curve calculated from the known atomic coordinates of the crystal structure of the RII tetra-tandemer using CRYSOL (red line) [34]. (**B**) Radial distribution function (RDF) obtained after IFT analysis of the scattering data, using data points starting from the first Guinier point until the Porod regimen (0.009<*q*<0.26 Å^−1^). Four maxima (**M1–4**) can be observed, which correspond to the centre of each domain of the RII tetra-tandemer.

**Figure 6:**
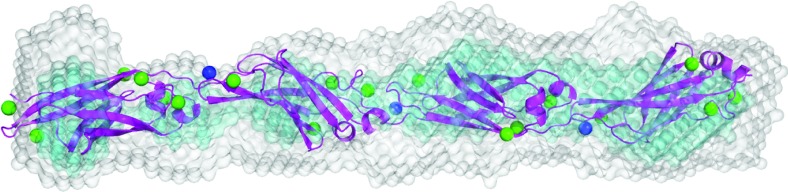
Aligned low-resolution dummy-atom model and crystal structure of RII tetra-tandemer in the presence of Ca^2+^ The averaged *ab initio* shape calculated from ten models (grey) represents the total volume occupied by the spread of all models (final goodness of fit, Rf=0.00084), with the filtered and most-populated volume represented in cyan and the crystal structure of RII tetra-tandemer in magenta. Ca^2+^ ions are represented as spheres.

To verify that the crystal structure is representative of the structure of the protein in solution, a low-resolution model was constructed from the experimental SAXS data using the *ab initio* modelling program DAMMIN [28]. DAMMIN uses an enclosed search volume of densely packed dummy atoms to reconstruct the shape of the protein in solution. Ten independent models were calculated and all provided a good fit to the experimental data (Figure 5A). The ten models were averaged using DAMAVER and no models in the set were rejected [30]. The resulting molecular shape of the *ab initio* model gives a good overlay with the crystal structure of the RII tetra-tandemer (Figure 6). Furthermore, evaluation of the atomic structure with the solution scattering data using CRYSOL also yields a good fit (Figure 5A), corroborating that the crystal structure is representative of the structure of the protein in solution (34).

## DISCUSSION

When the antifreeze activity of *Mp*AFP was first detected we suspected it might be localized to the periplasmic space of *M. primoryensis* [7]. The rationale was that an AFP in this location would bind and inhibit the growth of embryonic ice crystals arising from the extracellular environment before they could cause freezing damage to the bacterial cell. Subsequently, we realized that *Mp*AFP is a giant 1.5-MDa multidomain protein, and that its ice-binding domain (RIV) makes up only ~2% of the protein's mass [8,9]. The exceptionally large size and domain organization of *Mp*AFP is atypical of an AFP, which usually contains a single domain of mass 3–30 kDa [35]. This cast doubt on the primary function of *Mp*AFP being to help the bacterium resist freezing. Moreover, the domain architecture of *Mp*AFP and the presence of C-terminal RTX sequences are hallmarks of many large adhesion proteins. *Mp*AFP was detected on the outer surface of *M. primoryensis*, and is probably transported there using the type I secretion (TISS), since the C-terminal (RIV and RV) RTX repeats can potentially serve as the signal sequence for this pathway [9]. Based on these findings we speculated that *Mp*AFP is a surface adhesin that helps its host bacterium bind to ice.

**Figure 7:**
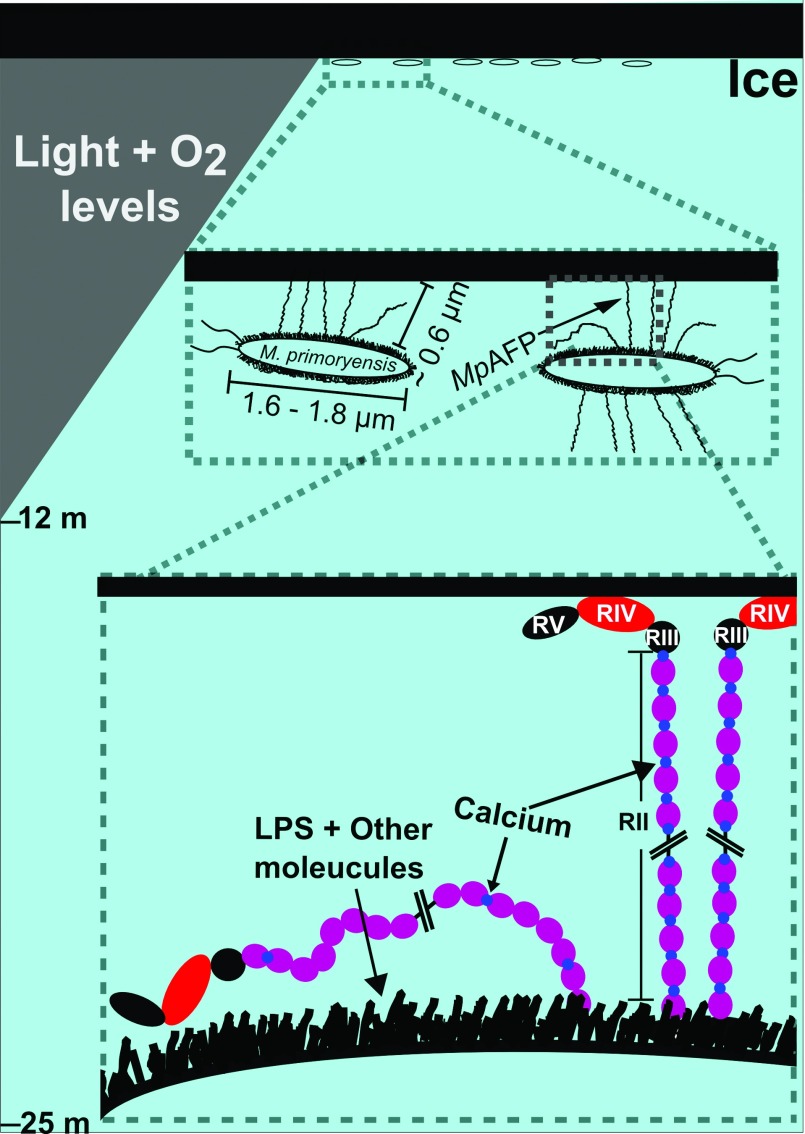
Hypothesized role of *Mp*AFP_RII in helping *M. primoryensis* bind to ice Cartoon representation of *M. primoryensis* in contact with the lower surface of lake ice represented by a series of views at increasing magnification from top to bottom. Top panel: oxygen content decreases with depth in the upper reaches of Ace Lake in Antarctica (0–12 m), whereas the lower reaches are anoxic (12–25 m). *M. primoryensis* is strictly aerobic and are positioned immediately under the ice. Middle panel: the bacteria's flagella are represented as two squiggles on one end of the bacterium; *Mp*AFP are represented by long filaments, some of which contact the ice. Lower panel: short thick lines on the exterior of the bacterium represent surface molecules such as proteins and polysaccharides other than *Mp*AFP. Inter-repeat Ca^2+^ that rigidify the junction regions linking each 104-aa repeat (purple sphere) are indicated by small blue spheres. Cross-hatched lines are used to represent the majority of RII repeats not shown in the diagram.

*M. primoryensis* was isolated from Ace Lake in eastern Antarctica. The surface of this brackish lake is covered with ice (1–2 m thick) for approximately 11 months of the year, which maintains the temperature of the water column between −1 and 1°C [36,37]. Since the accumulation of snow on the lake ice further attenuates light to the water below, only those phytoplankton and other photosynthetic micro-organisms that occupy a position close to the top of the water column will flourish in this limited photic zone. Given that ice on the lake surface prevents the wind-driven mixing of the lake water, the oxygen content of Ace lake is highest in its upper reaches (0–12 m), while the lower part of the lake is anoxic (12–25 m) (Figure 7). We have hypothesized that *M. primoryensis* uses *Mp*AFP to bind the underside of ice covering the lake surface [9]. This locates the strictly aerobic bacterium in a favourable position where it can gain access to oxygen and other nutrients from the nearby photosynthetic micro-organisms without expending energy. Bioinformatic analyses have suggested the Gram-negative *Shewanella frigidamarina* isolated from the Antarctic sea ice contains a different ice-binding protein linked to BIg domains [38]. It is possible that different micro-organisms have evolved similar envirotactic strategies to remain in favourable environments. A novel mechanism to this end has been proposed for non-motile diatoms isolated from the overlying ice of the Laurentian Great Lakes. It was hypothesized that the diatoms might associate with frazil ice for the subsequent recruitment to ice near the lake surface, where a better light climate is present [39].

The ice-binding RIV domain of *Mp*AFP is the logical region to bind the host bacterium to ice [9]. However, the role of the large repetitive RII in this bacterium–ice interaction was unclear due to a lack of detailed structural information. The non-ice-binding RII contains roughly 120 tandem copies of identical 104-aa repeats. Previously, bioinformatics and X-ray crystallographic analyses indicated that RII has many attributes that link it to adhesion proteins. RII is found on the exterior Gram-negative bacterial cell envelope and each individual RII repeat folds as a Ca^2+^-dependent Ig-like β-sandwich. Here, we determined the crystal and solution structures of the RII tetra-tandemer, which displays RII tetra-tandemer repeats linked into an extended, ‘train-like’ structure. As the RII repeats are identical, the knowledge gained from the crystal structure of the RII tetra-tandemer can be applied to predict the overall architecture of the ~120 tandem RII repeats, which likely forms a long chain of compact domains. This is reminiscent of the type I pilus adhesin found in many Gram-negative bacteria. A type I pilus typically contains 500–3000 Ig-like subunits (similar to *Mp*AFP_RII) that helps project the adhesive tip domain (such as *Mp*AFP_RIV) up to 2 μm away from the bacterial cell surface [40]. This property of the type I pilus serves to reduce the charge-driven repulsive force between the host bacterium and its target cell, by keeping a sufficient distance between the cell-surfaces. *Mp*AFP may mimic the adhesion mechanism of the type I pilus in binding *M. primoryensis* to ice (Figure 7). The Ca^2+^-rigidified linker regions could potentially extend the tandem Ig-like domains of RII into a ~0.6 μm rod-like structure. This length between the ice-adhesive RIV and the bacterium's cell surface could be critical. The exterior of the Gram-negative bacterial cell envelope is covered with a layer of lipopolysaccharide and other macromolecules. Therefore it is perhaps necessary for RII to help RIV protrude from the surface milieu to be able to efficiently interact with ice. The lipopolysaccharide layer also confers to the bacterial outer membrane an overall negative charge. *Mp*AFP_RII is rich in negatively charged acidic residues (18% Asp+Glu), and contains no Lys or Arg [12]. The acidic residues of RII not only help coordinate Ca^2+^ to stabilize the protein's fold, but also may be repelled from the negatively charged cell surface for better extension of the ice-binding domain.

A semi-rigid, extended RII could help the ice-binding RIV sweep over a large area to contact ice. The ice-bacterium interaction is unlikely to be permanent. We have observed that monomeric AFPs are overgrown by, and included into, ice [41] but larger structures like phage displaying AFP on their coat proteins are sheared off the ice surface (M. Tomczak and P.L. Davies, unpublished work). Since bacteria are even larger than the phage, they too are unlikely to be included into the ice. However, if some adhesin contacts are sheared off by the growing ice there are many others on the bacterial surface that could resecure the bacteria to the ice.

The brackish-water of Ace Lake has high salinity, and is rich in divalent cations such as Ca^2+^ (3–7 mM) and Mg^2+^ (35–85 mM) [36]. *Mp*AFP_RII protomers require roughly 10 molar equivalents of Ca^2+^ to be fully structured [12]. The ice-binding RIV also requires the presence of millimolar Ca^2+^ for folding. The Ca^2+^-dependency of *Mp*AFP domains helps explain how such a giant protein of 1.5-MDa is secreted via TISS. Ca^2+^ is normally present in sub-micromolar concentrations in the bacterial cytosol. Therefore the large *Mp*AFP is likely secreted as a long but unfolded chain of polypeptide, and only folds upon entering the extracellular brackish lake water, where Ca^2+^ is abundant. The Ca^2+^-stabilization of *Mp*AFP's structure may also protect the protein against proteolysis by extracellular proteases. It has been shown that *Mp*AFP retains its ice-binding activity in the presence of Ca^2+^ after it was incubated with trypsin for up to 6 days. In contrast, in the absence of Ca^2+^, the activity was completely lost by 30 min [7].

Recent advances in genome sequencing have helped identify many large repetitive adhesion proteins in bacteria. Well-characterized examples include the cell-wall-associated adhesion protein (Ebh) from the Gram-positive *Staphylococus aureus*; the large RTX adhesins found in many Gram-negative bacteria, including biofilm-associated proteins of LapA and LapF from *P. putida*; and epithelial adhesin SiiE from *S. enterica*. However, the extreme repetition within the extender domains, which can be identical even at the DNA level, has caused difficulties in sequencing the ORFs (open reading frames) of some RTX adhesins. As a result, these large ORFs are often improperly annotated and appear as two separate contigs in the databases [42]. Thus many of the large RTX adhesins remain to be described, and their importance in biofilm formation and pathogenesis are yet to be fully realized.

In conclusion, we have reported the crystal and solution structures of four tandem Ig-like repeats of the extender domain of a 1.5 MDa ice-binding RTX adhesin from an Antarctic bacterium. This work is relevant to many other large repetitive proteins, especially those of the RTX adhesins that facilitate infections by animal pathogens such as *Salmonella*, *Vibrio* and *Pseudomonas*.

## Online data

Supplementary data
