# Supplementary material for: Ca2+-stabilized adhesin helps an Antarctic bacterium reach out and bind ice
Source: Biosci Rep. 2014 Jul 4;34(4):e00121. doi: 10.1042/BSR20140083 (PMC4083281; doi:10.1042/BSR20140083)
Supplement: Supplementary data [file bsr034e121add.pdf]

Biosci. Rep. (2014) / **34** / art:e00121 / doi 10.1042/BSR20140083

Original Paper

OPEN ACCESS

Bioscience Reports

www.bioscirep.org

Bioscience Reports

SUPPLEMENTARY DATA

Ca^{2+}-stabilized adhesin helps an Antarctic bacterium reach out and bind ice

Tyler D. R. VANCE*, Luuk L. C. OLIJVE†, Robert L. CAMPBELL*, Ilja K. VOETS†, Peter L. DAVIES* and Shuaiqi GUO*[1]

*Protein Function Discovery Group and the Department of Biomedical and Molecular Sciences, Queen's University, Kingston, Ontario, Canada
†Laboratory of Macromolecular and Organic Chemistry and Institute for Complex Molecular Systems, Department of Chemical Engineering and Chemistry, Eindhoven University of Technology, Eindhoven, The Netherlands

SAXS

Guinier analysis and Kratky plot of the RII tetra-tandemer

A Guinier analysis was used to obtain the radius of gyration (R_g) and molecular weight ($M_{w,\mathrm{SAXS}}$) of the protein construct. For monodisperse globular proteins, the Guinier approximation (valid for $qR_g \leqslant 1.3$) gives an estimation of their size, using

$$I(q) = I_0 e^{\frac{1}{2Rg^2q^2}}$$

with the radius of gyration, R_g, and the forward scattering intensity, I_0, for I at $q = 0$. The R_g and I_0 were determined from the slope and y-intercept of the Guinier plot $\ln(I(q))$ versus q^2. The scattering intensity at zero angle can be used to calculated the molecular weight of the protein using

$$M_{w,\mathrm{SAXS}} = I(0)\frac{Nsv}{c(\Delta pv)^2} \qquad \text{(S1)}$$

with the molecular weight M_w,SAXS in g/mol, the forward scattering intensity I_0 in cm^{-1}, concentration c in $\mathrm{g/cm}^3$, Avogadro's number N_{av}, the scattering length density difference $\Delta\rho$ in cm^{-2} ($\rho_{\mathrm{protein}} - \rho_{\mathrm{H2O}}$, where $\rho_{\mathrm{protein}} = 1.25\times10^{11}$ cm^{-2} and $\rho_{\mathrm{H2O}} = 9.44\times10^{10}$ cm^{-2}) and the partial specific volume of the protein in solution $\bar{v} = 0.734$ in cm^3/g [43].

A Guinier analysis is an important check of monodispersity and/or aggregation, which can be observed by non-linearity in the Guinier plot due to an up- or downturn at the lower q-values.

The molecular weight M_w,$_{\mathrm{SAXS}}$ determined from the forward scattering intensity I_0 is in good agreement with the theoretical value for the RII tetra-tandemer in the presence of EDTA, while M_w,$_{\mathrm{SAXS}}$ seems slightly overestimated in the presence of calcium ($M_{w,\mathrm{calc}} = 42.6$ kDa versus M_w,$_{\mathrm{SAXS}} = 52.6$ kDa). As there is no evidence for aggregation or non-negligible protein–protein interactions (all Guiner plots are linear), we tentatively attribute this discrepancy to a deviation of the specific volume of the tetra-tandemer from $\bar{v} = 0.734$ cm^3/g.

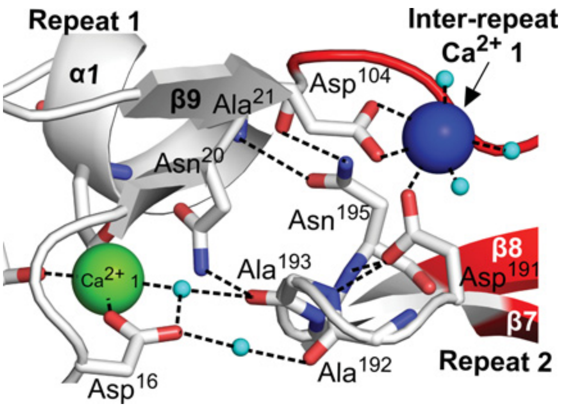

Figure S1 Enlarged view of the linker region between Repeats 1 and 2
The colour scheme is the same as in Figure 3(E).

Structural data are available in the Protein Data Bank under the accession number of 4P99.

[1] To correspondence should be addressed (email guo.shuaiqi@queensu.ca).

© 2014 The Author(s) This is an Open Access article distributed under the terms of the Creative Commons Attribution Licence (CC-BY) (http://creativecommons.org/licenses/by/3.0/) which permits unrestricted use, distribution and reproduction in any medium, provided the original work is properly cited.

Table S1 Parameters obtained from the Guinier analysis of the experimental data of RII tetra-tandemer, with c = concentration, $M_{w,calc}$ = theoretical molecular mass calculated from the amino acid sequence, I_0 = forward scattering intensity extrapolated to zero angle, R_g = radius of gyration, $M_{w,saxs}$ = molecular mass determined by SAXS using eqn S1.

	c (mg/ml)	$M_{w,calc}$ (kDa)	I_0 (cm^{-1})	R_g (Å)	$M_{w,SAXS}$ (kDa)
$CaCl_2$	5	42.6	0.222 ± 0.001	44.9 ± 0.5	52.6
EDTA	5	42.6	0.185 ± 0.003	36.2 ± 0.1	43.9

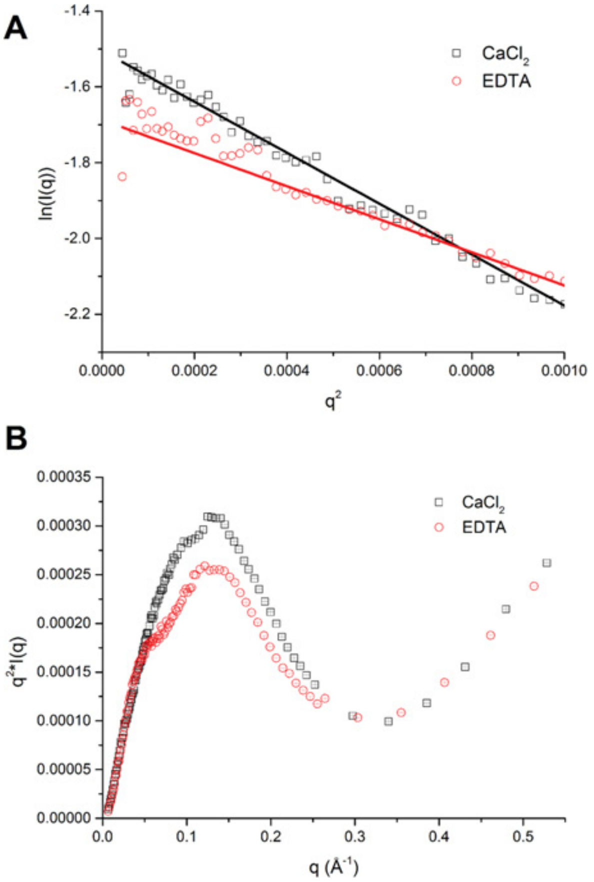

Figure S2 Guinier and Kratky representation of SAXS data
(**A**) Guinier plot of the experimental SAXS data (open symbols) and fits of the Guinier approximation. (**B**) Kratky representation of the experimental data [$q^2 * I(Q)$ versus q].

Received 29 May 2014; accepted 3 June 2014

Published as Immediate Publication 3 June 2014, doi 10.1042/BSR20140083

© 2014 The Author(s) This is an Open Access article distributed under the terms of the Creative Commons Attribution Licence (CC-BY) (http://creativecommons.org/licenses/by/3.0/) which permits unrestricted use, distribution and reproduction in any medium, provided the original work is properly cited.
